# Cardioprotective effects of Fenugreek (*Trigonella foenum-graceum*) seed extract in streptozotocin induced diabetic rats

**DOI:** 10.34172/jcvtr.2021.01

**Published:** 2021-01-13

**Authors:** Soleyman Bafadam, Maryam Mahmoudabady, Saeed Niazmand, Seyed Abdolrahim Rezaee, Mohammad Soukhtanloo

**Affiliations:** ^1^Department of Physiology, Faculty of Medicine, Mashhad University of Medical Sciences, Mashhad, Iran; ^2^Applied Biomedical Research Center, Mashhad University of Medical Sciences, Mashhad, Iran; ^3^Immunology Research Center, Inflammation and Inflammatory Diseases Division, Mashhad University of Medical Sciences, Mashhad, Iran; ^4^Department of Clinical Biochemistry, Faculty of Medicine, Mashhad University of Medical Sciences, Mashhad, Iran

**Keywords:** Diabetes, Fenugreek Seed, Cardiomyopathy, Oxidative Stress, Apoptosis

## Abstract

***Introduction:*** Inadequate control of diabetes mellitus (DM) leads to considerable cardiovascular implications like diabetic cardiomyopathy (DCM). Cardiomyocyte apoptosis is one of the main mechanisms of DCM pathogenesis associated with hyperglycemia, oxidative stress, inflammation, hyperlipidemia and several other factors. *Trigonella foenum-graecum* (Fenugreek) has been long used as a traditional medicine and has many therapeutic effects, including anti-diabetic, anti-hyperlipidemia, anti-inflammatory and anti-oxidant properties. The current study aimed to investigate cardioprotective effects of fenugreek seed on diabetic rats.

***Methods:*** Diabetes was induced in forty-two male rats by injection of streptozotocin (STZ) (60 mg/ kg). Diabetic animals were treated with three different doses of fenugreek seed extract (50, 100 and 200 mg/kg) or metformin (300 mg/kg) for six weeks by gavage. Nondiabetic rats served as controls. Glucose, cholesterol, and triglycerides levels were measured in the blood samples, and oxidative stress markers as well as gene expression of *ICAM1* , *Bax* and *Bcl2* were assessed in the cardiac tissues of the experimental groups.

***Results:*** Diabetic rats exhibited increased serum glucose, cholesterol and triglycerides levels, elevated markers of oxidative stress thiobarbituric acid–reacting substances (TBARS) levels , total thiol groups (SH), catalase (CAT) and superoxide dismutase (SOD) activity, and enhanced apoptosis cell death (ratio of Bax/Bcl2). Fenugreek seed extract considerably improved metabolism abnormalities, attenuated oxidative stress and diminished apoptosis index.

***Conclusion:*** Our study suggests that fenugreek seed may protect the cardiac structure in STZ-induced diabetic rats by attenuating oxidative stress and apoptosis.

## Introduction

Today, diabetes mellitus (DM) with its increasing incidence is considered the most common metabolic disorder in the world. DM has several long-term complications on various body organs such as brain, eyes, cardiovascular system, and kidneys.^1,2^

DM can cause numerous cardiovascular diseases (CVDs) by implicating large arteries and coronary arteries, which ultimately lead to ischemia, stroke, and peripheral vascular diseases (PVD). In addition to these vascular complications, diabetes can cause a type of cardiomyopathy called “diabetic cardiomyopathy (DCM)”. DCM is one of the DM complications of the heart tissue regarded as diabetes-associated changes in the function and structure of the myocardium that is not directly associated with other confounding factors such as coronary artery disease (CAD), atherosclerosis, and hypertension.^3^ Several factors are involved in the pathogenesis of DCM such as increased activity of renin angiotensin aldosterone system (RAAS), hyperglycemia, increased production of advanced glycation end products (AGEs), increased consumption of fatty acids by cardiomyocytes, insufficiency of calcium hemostasis by cardiomyocytes, autophagy, mitochondrial failure, endoplasmic reticulum stress, cardiomyocyte apoptosis and necrosis and myocardial fibrosis.^4^ Cardiac cell apoptosis is one of the main mechanisms of DCM pathogenesis associated with hyperglycemia, oxidative stress, inflammation, hyperlipidemia and several other factors.^5^ In response to myocardial cell death, hypertrophy of remaining cardiomyocytes and fibrosis replacement is implicated in structural cardiac muscle remodeling and dysfunction. Oxidative stress is the damage caused by reactive oxygen substance (ROS) due to the imbalance of free radicals and antioxidants level in the body. In this case, generation of ROS is higher than activity of free radical’s scavenger enzymes such as CAT, SOD glutathione reductase (GR), and glutathione peroxidase (GPx), as well as non-enzymatic antioxidants like glutathione (GSH) and thiol groups (SH). Lipid peroxidation is one of the main productions of free radicals’ activity, which can destruct cellular membranes, leading to toxic effects and cell death. Level of thiobarbituric acid reactive substances (TBARS) is a marker for lipid peroxidation^6^. Hyperglycemia-induced oxidative stress plays a critical role in apoptosis induction as well as development and progression of DCM.^6,7^ In this regard, overexpression of the pro-apoptotic *Bax* gene, downexpression of the anti-apoptotic *Bcl2* gene as well as overexpression of the *ICAM1* gene expression indicate increased apoptosis in STZ-diabetic myocardium, and have implications for cardiomyocyte apoptosis and the pathogenesis of diabetic cardiomyopathy.^8,9^

However, an agent possessing both hypoglycemic and antioxidant activities could be useful to prevent cardiomyocyte apoptosis and DCM.

*Trigonella foenum-graceum* (Fenugreek) herb has long been used in traditional medicine and has many therapeutic benefits. The fenugreek seeds contain the alkaloid trigonelline with tannic acid, mucilage, fixed and volatile oils, a bitter extractive, and yellow coloring matter, as well as steroidal saponins such as diosgenin, gitogenin and a trace of trigogenin and vitamin A. In addition, this seed is a rich source of fiber and proteins.^10,11^

Several reports demonstrate the therapeutic effects of fenugreek plant such as hypoglycemic, analgesic, anti-atherosclerotic, anti-inflammatory, antispasmodic, anticancer, cardioprotective, blood cholesterol-lowering, lipid-lowering, labor induction, promoting milk flow, antibacterial, and anti-worm.^12,13^

Many former studies indicated the antidiabetic effect of fenugreek and showed that fenugreek seed extract had high potential in removing free radicals and inhibiting oxidative stress; therefore, it is widely used to treat DM.^14,15^ Moreover, its cardioprotective effect in diabetic heart has been shown recently^16^, but its effects on cardiac apoptosis have not been investigated.

In this regard, this study aimed to investigate effects of fenugreek seeds hydro alcoholic extract on heart tissue oxidative stress and apoptosis as well as blood levels of glucose, triglyceride and cholesterol in STZ-induced diabetic rats.

## Material and methods

### Plant material and preparation of seed extract

*Trigonella foenum-graceum* seeds were harvested from Neyshabour city, Khorasan province, Iran. The seeds were identified by botanists in the Herbarium of Ferdowsi University of Mashhad, (Voucher NO. 13248).

One kilogram of seeds was grinded and soaked in 10 liters of hydroalcoholic solution (70% ethanol, 30% water) for 72 hours at 37 °C on a shaker incubator. The extraction solution was subsequently filtered and evaporated under vacuum at 40°C until it was evaporated. To obtain doses of 50, 100 and 200 mg/kg, the dried extract was dissolved in distilled water^11^. The extract yield was 20% on average.

### Animals treatment

Male Wistar rats weighing 250 ± 30 g were provided from the Animal Center of Mashhad University of Medical Sciences. Standard cages were used to keep animals, two or three in each cage, in a room under controlled temperature (22±1 ^o^C) and dark–light cycles with free access to standard laboratory diet and drinking water.

We used the STZ-induced diabetes model in rats. Junod et al. ^17^ explained the diabetogenic effects of STZ and revealed that STZ was able to destruct pancreatic islet β-cells selectively. Because of this action, the animals experienced insulin deficiency, hyperglycemia, polydipsia, and polyuria, all of which are the characteristics of human type 1 diabetes mellitus. ^18^

Moreover, considering previous reports, DCM was foresighted after six weeks in this model. ^19^

STZ (60 mg/kg, i.p.) was injected at a single dose to induce diabetes. The development of the diabetes was confirmed by assessing blood glucose levels in the tail blood samples of the 12-hour fasted rats. The rats having the blood glucose level≥250 mg/dL were considered diabetic.^20^

The animals were randomly divided into six groups (7 rats in each group):

Diabetic, metformin treated diabetic rats, control, and three groups of fenugreek seed extract treated diabetic rats (Fen 50, Fen 100, and Fen 200). Normal saline was orally administered by gavage to the control and diabetic groups. In this regard, the metformin group received 300 mg/kg of drug according the previous study^21^, and fenugreekseed extract (50, 100, and 200 mg/kg) was daily given to the Fen group by gavage for 6 weeks; these doses were selected based on the values used in previous studies.^22^

After the treatment period, the fasted rats were anesthetized and euthanized by guillotine decapitation. The animals’ blood samples were collected from heart chambers and centrifuged at 3000 rpm, and the separated serum was maintained at -20 ^o^C.

The animal chest was opened, and the heart tissue was rapidly dissected out on ice, part of left ventricle was quickly placed in the RNA later solution to assess its gene expression, and the rest of heart tissue was placed in -70 ^o^C to determine oxidative stress markers.

### Plasma glucose, cholesterol and triglyceride concentration

Serum fasting blood glucose concentrations were measured before STZ injection, on the third day and in the sixth week (45th day).

Cholesterol and triglyceride concentrations were evaluated at the end of the protocol, and calorimetry diagnostic kits (Pars Azmoon Tehran, Iran) were used to measure the above parameters.

### Heart tissue oxidative stress assessment

To analyze the tissue, weighed amounts of the frozen tissues were homogenized in a suitable buffer using a microcentrifuge tube homogenizer. TBARS levels, SH, CAT and SOD activity were measured in the heart tissue oxidative stress.

### Measurement of lipid peroxidation

TBARS levels as the index of lipid peroxidation were measured according to the previous protocol.^23^

The degree of lipid peroxidation was examined by measuring the malondialdehyde (MDA) level. MDA is conjugated to thiobarbituric acid (TBA) in TBARS, and produces a stable red color with maximum absorption at 535 nm. According to this method, 1 mL of homogenates was combined with 2 mL of trichloroacetic acid (TCA) –TBA - hydrochloric acid (HCl), and heating the solution for 40 minutes was carried out in a boiling water bath. At the end of cooling, the mixture was centrifuged at 1000 g for 10 minutes, and the sample absorbance was read at 535 nm. Using an extinction coefficient of 1.56 × 10^5^ cm^−1^/M^−1^, the concentration was expressed as nmol of TBARS per gram of tissue weight.

### Measurement of protein thiol (SH) groups

Using DTNB (2, 2’-dinitro- 5, 5’-dithiodibenzoic acid) as the reagent, the total thiol content was estimated. DTNB has low absorbance, but when it reacts with SH groups, it produces a yellow complex having a peak absorbance at 412 nm. In brief, 1 mL Tris-EDTA buffer (pH=8.6) was added to 50 µL of tissue homogenates, and absorbance of the sample was read at 412 nm against the Tris-EDTA buffer alone (A1). Afterward, 20 mL DTNB reagents (10 mM in methanol) were added to the solution, and after 15 minutes (kept at room temperature), the absorbance of the sample was re-read (A2). The DTNB reagent absorbance was also read as a blank (B). Total thiol concentration (mM) was measured by the following equation:

Total thiol concentration (mM) = (A2-A1-B) × 1.07 / (0.05 × 14,150). ^24^

### Determination of anti-oxidant enzymes

SOD activity was specified based on Madesh and Balasubramanian’s method to measure the ability of the enzyme to inhibit reduction of the tetrazolium dye, 3- 4,5-dimethylthiazol-2-yl)-2,5-dimethyltetrazolium bromide (MTT) via superoxide. The absorbance was measured at 570 nm in a spectrophotometer. One SOD unit was regarded as the enzyme amount necessary to inhibit MTT reduction by 50%. ^25^

Catalase decomposes H_2_O_2_ and produces water and molecular oxygen.

Catalase activity was assayed by Aebi’s method.^26^ The enzymatic activity measurement of catalase was performed by monitoring the declined absorbance at 240 nm owing to H_2_O_2_ decomposition for 1 minute.

### RNA extraction and cDNA synthesis

Based on the manufacturer’s instructions, total RNA was isolated from the homogenized rat heart by applying reagent of YTzol Pure RNA solution (Yekta Tajhiz Azma, Iran). The samples’ quality and quantity were spectrophotometrically indicated by applying the A260/A280 ratio (Nano Drop ND-1000, Thermo Scientific, Delaware, USA), and they were assessed through visualizing ribosomal bands 28S and 18S with ethidium bromide after electrophoretic isolation over formaldehyde denaturing 1.2 % agarose gel. The first-strand cDNA was reverse-synthesized by the RevertAid^TM^ First-Strand cDNA Synthesis Kit (Fermentas, Germany), along with random hexamer primers according to the manufacturer’s instructions using BioRad C1000 Touch^TM^ Thermal Cycler (Bio-Rad Laboratories, USA).

### Quantitative Real-Time PCR (qPCR)

The primers of the quantitative real time PCR Syber Green method were designed for complete cDNA of* Bcl-2, Bax*, *ICAM1* and* β-actin* based on the sequence data on NCBI databases by utilizing the Beacon Designer software (PREMIER Biosoft International, Palo Alto, CA; version 7). Specificity of the primers was examined by BLAST analysis (NCBI). A quantitative real-time PCR was carried out by SYBR Green Master Mix (Takara, Japan). The primers’ specificity and reliability were approved via performing endpoint PCR and amplicon sequencing through DNA sequencing by applying biosystems (SEQLAB, Göttingen, Germany). Table 1 summarizes the designed primers. β-actin was used as the housekeeping gene to normalize the data.^27^ The relative two standard curves real-time PCR Syber Green method was used to analyze gene expressions in a LightCycler (Roche Diagnostics, Mannheim, Germany). The data were analyzed using 6-point 10-fold serial dilution standard curves for related and reference genes. Standard curves were plotted for reference and target genes. Afterward, the LightCycler software was employed for data analysis. The relative quantity of *Bcl-2, Bax* and *ICAM1* gene expressions was normalized to the β-actin mRNA relative quantity and showed as the gene expression index. Relative gene expression (fold changes) was calculated using the 2^−ΔΔCT^ method.^28^

**Table 1:** Primers designed for semi-quantitative real-time polymerase chain reaction

**Gene**	**Primers sequences**
*Bcl-2*	Forward: 5'- AGG ATA ACG GAG GCT GGG ATG-3' Reverse: 5'- CTC ACT TGT GGC CCA GGT ATG-3'
*Bax*	Forward: 5'-CAT CAG GGT TTC ATC CAG GAT C-3' Reverse: 5'- CCA CAT CAG CAA TCA TCC TCT G-3'
*ICAM1*	Forward: 5'-TCT TGC GAA GAC GAG AAC CTC-3' Reverse: 5'-GCT CTG GGA ACG AAT ACA CAG-3'
*β-actin*	Forward: 5'-CCC GCG AGT ACA ACC TTC T-3' Reverse: 5'- CCA TCA CAC CCT GGT GCC TA-3'

## Statistical analysis

Data analyses were conducted using the SPSS 20.0 statistical package (IBM SPSS Inc., USA). All the values were showed as mean ± standard error of mean (SEM). Additionally, for statistical assessment, analysis of variance (ANOVA) followed by Tukey test analysis was conducted. The significance level was considered *P* < 0.05.

## Results

### Plasma glucose, cholesterol, and triglycerides

**Figure 1:**
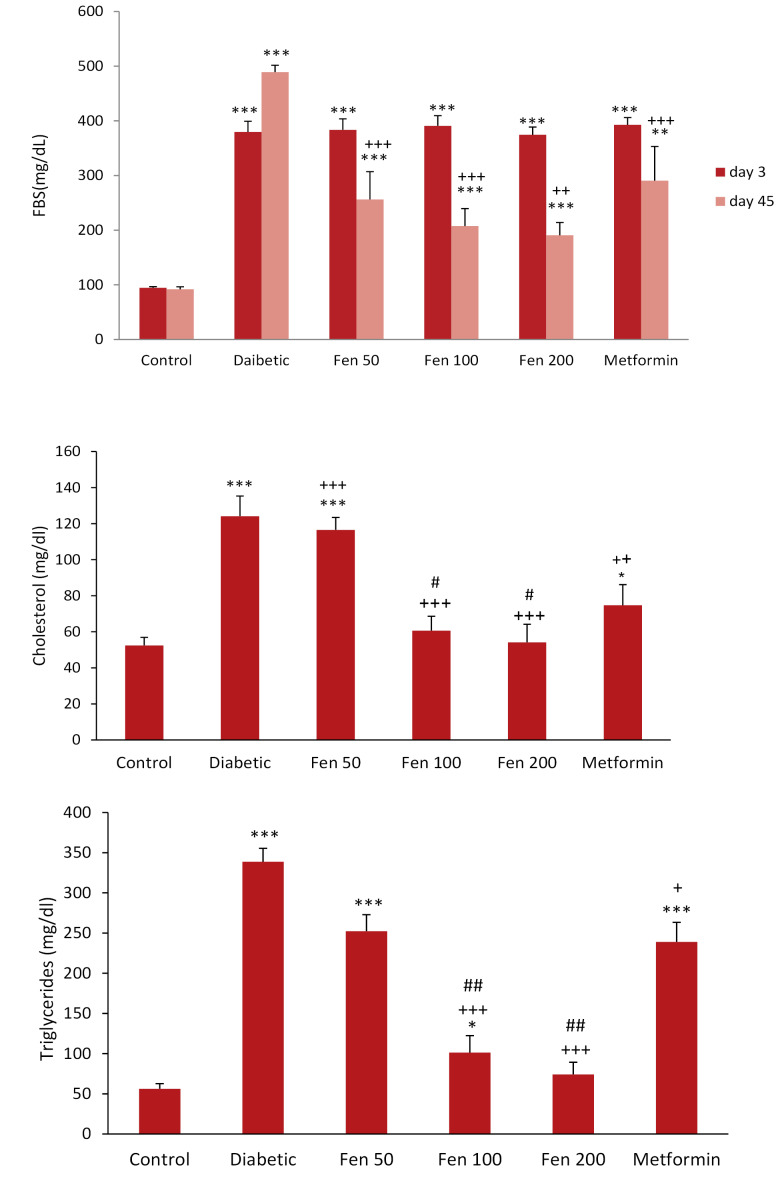
Serum concentrations of (A) fasting blood sugar (FBS) on days 3 and 45, (B) cholesterol and (C) triglycerides on day 45, in all experimental groups. Results are expressed as mean ± SEM, n = 7, statistical assessment was conducted using analysis of variance (ANOVA) followed by Tukey’s test analysis. “*P < 0.05, **P < 0.01, ***P < 0.001 vs. Control group”. “+*P* < 0.05, ++*P* < 0.01, +++*P* < 0.001 vs. the diabetic group”. “#*P* < 0.05, ##*P* < 0.01 vs. Metformin group”. Fen 50: Fenugreek seed extract 50 mg/kg, Fen 100: Fenugreek seed extract 100 mg/kg, Fen 200: Fenugreek seed extract 200 mg/kg

Three days after STZ injection, serum glucose levels in all injected groups were significantly higher than in the control group (*P* < 0.001 for all). Six weeks (Day 45) after administration of the fenugreek seed extract, the blood glucose levels significantly reduced in all extract treated groups (*P* < 0.001 for all) than in the diabetic group. Metformin also decreased levels of glucose compared to the diabetic group (*P* < 0.01), (Figure 1A).

After the treatment period, total blood cholesterol and triglycerides levels in the untreated diabetic, Fen 50 and metformin groups were significantly higher than in the control group (*P* < 0.05 and *P* < 0.001). In addition, the triglycerides level in Fen 100 was significantly higher than in the control group (*P* < 0.05), while, these amounts were significantly decreased in Fen 100, Fen 200 and metformin groups than in the untreated diabetic group (*P* < 0.05 to *P* < 0.01). Furthermore, these lipid levels in Fen 100 and Fen 200 groups were significantly lower than in the metformin group (*P* < 0.05 and *P* < 0.01), (Figure 1B, 1C).

### The effects of fenugreek seed extract on oxidative stress state

**Figure 2:**
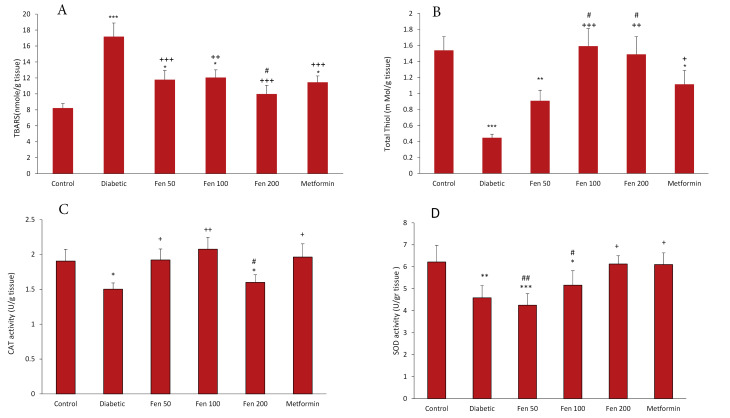
Heart tissue concentrations of (A) Thiobarbituric acid reactive substances (TBARS) and (B) thiol and activities of (C) catalase and (D) superoxide dismutase (SOD) in all the experimental groups. Results are expressed as mean ± SEM, n = 7, statistical assessment was conducted using analysis of variance (ANOVA) followed by Tukey’s test analysis. “*P < 0.05, **P < 0.01, ***P < 0.001 vs. Control group”. “+*P* < 0.05, ++*P* < 0.01, +++*P* < 0.001 vs. the diabetic group”. “#*P* < 0.05, ##*P* < 0.01 vs. Metformin group”. Fen 50: Fenugreek seed extract 50 mg/kg, Fen 100: Fenugreek seed extract 100 mg/kg, Fen 200: Fenugreek seed extract 200 mg/kg

TBARS levels in the heart tissue of the diabetic group were higher than those of the control group, whereas the thiol concentration was significantly lower in this group than in the control group (*P* < 0.001 for both). Treatment with all doses of the extract and metformin decreased TBARS levels in the heart tissue compared to the diabetic group (*P* < 0.01 to *P* < 0.001). Moreover, in the Fen 200 group, it was significantly lower than that in the metformin group (*P* < 0.05). Thiol concentration was significantly increased in Fen100, Fen 200, and metformin groups than in the diabetic group (*P* < 0.05 to *P* < 0.001), and higher in Fen 100 and 200 groups than in the metformin group (*P* < 0.05) (Figure 2A, 2B).

Heart tissue catalase and SOD enzyme activities in the diabetic group were at a significantly lower level than that in the control group (*P* < 0.05 to *P* < 0.01), while catalase enzyme activity in Fen 50, Fen 100 and metformin treated groups was at a significantly higher level than that in the diabetic group (*P* < 0.05 to *P* < 0.01). SOD activity was significantly higher in Fen 200 and metformin treated groups than in the diabetic group (*P* < 0.05 for both). Catalase activity in Fen 200 and SOD activity in Fen 50 and Fen 100 were significantly lower than the ones in the metformin group (*P* < 0.05 to *P* < 0.01). (Figure 2C, 2D).

### Gene expression and apoptosis index

**Figure 3:**
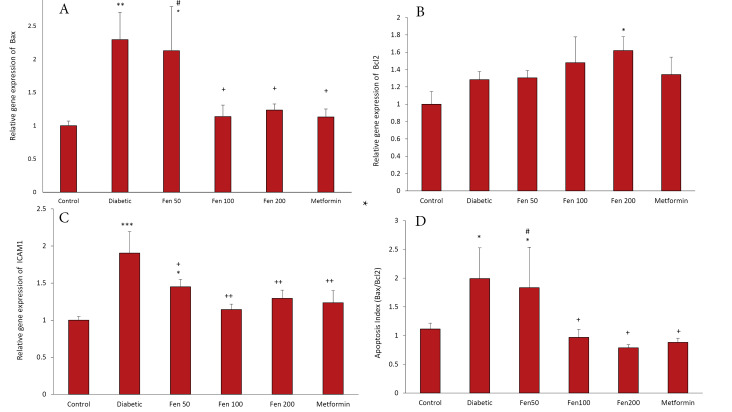
Relative gene expression (quantified gene expression by comparing target transcripts and reference genes) of (A) *Bax*, (B) *Bcl2* (C) *ICAM1* and (D) apoptosis index (Bax/Bcl2 ratio) in heart tissue of all the experimental groups. Results are expressed as mean ± SEM, n = 7, statistical assessment was conducted using analysis of variance (ANOVA) followed by Tukey test analysis. “*P < 0.05, **P < 0.01, ***P < 0.001 vs. Control group”. “+*P* < 0.05, ++*P* < 0.01 vs. the diabetic group”. “#*P* < 0.05 vs. Metformin group”. Fen 50: Fenugreek seed extract 50 mg/kg, Fen 100: Fenugreek seed extract 100 mg/kg, Fen 200: Fenugreek seed extract 200 mg/kg

The results of quantitative real-time PCR indicated that left ventricle *Bax* and *ICAM1* relative gene expressions in the diabetic group were significantly higher than those in the control group (*P* < 0.01 to *P* < 0.001) (Figure 3A, 3C).

Treatment with Fen 100, Fen 200, and metformin significantly attenuated *Bax* and *ICAM1* relative gene expressions in comparison to the untreated diabetic group (*P* < 0.05 to *P* < 0.01). In the Fen 50 group compared to the diabetic group, *ICAM1* relative gene expression was reduced (*P* < 0.05) and *Bax* relative gene expression in the Fen 50 group was significantly higher than that in the metformin group (*P* <0.05) (Figure 3A, 3C).

The left ventricle *Bcl2* relative gene expression of the diabetic group did not significantly change compared to the control group (*P* < 0.05) and showed augmentation in Fen 100, Fen 200, and metformin groups in comparison to the diabetic group; however, this amelioration was significant only in the Fen 200 group (*P* < 0.05) (Figure 3B).

The apoptosis index (Bax/Bcl2 ratio) in diabetic and Fen 50 treated groups was significantly higher than in the control group (*P* < 0.01). Treatment with the fenugreek extract at doses of 100 and 200 and metformin significantly decreased the apoptosis index in comparison to the diabetic group (*P* < 0.05). However, this index was significantly higher in Fen 50 than in the metformin group (*P* <0.05), but it did not show any significant difference in Fen 100 and Fen 200 groups than in the metformin group (Figure 3D).

## Discussion

Numerous factors are implicated in the pathogenesis of DCM, hyperglycemia, oxidative stress and apoptosis play crucial roles in the development and progression of this implication.^6^

We observed that hyperglycemia and hyperlipidemia, which were obvious in diabetic rats, were significantly reduced in fenugreek seed extract and metformin treated groups, as in previous studies, these beneficial effects of fenugreek were emphasized.^29,30^ Our observation demonstrated that the beneficial effect of high doses of fenugreek on hyperlipidemia could be better than that of metformin.

Several mechanisms are suggested for the hypoglycemic effect of fenugreek seed; 4 hydroxyisoleucine content of fenugreek seed increases cell glucose uptake by increasing Glut-4 on the cell membrane surface, polyphenol component of fenugreek and cuerestin can increase insulin sensitivity by enhancing phosphorylation of tyrosine kinase and improving insulin signaling.^31,32^ Furthermore, some mechanisms responsible for the anti-hyperlipidemic property of fenugreek play their roles in this regard; it contains diosgenin that could change intracellular calcium concentration by which regulates membrane binding protein and in turn increases lipid transportation into the cells and decreases tissue fatty acids.^33^

Moreover, one of the components of fenugreek seed is pectin fibers fermented by iliac bacteria to short chain fatty acids such as butyric acid, propionate and acetate, thereby decreasing cholesterol synthesis.^34^

Oxidative stress plays a prominent role in the pathogenesis of DCM by causing myocardial damage at molecular and cellular levels, leading to cardiac structural and functional abnormalities.^6^

The present study data indicated that the heart tissue oxidative stress was higher in the diabetic group than in the control group as confirmed by the increase of the TBARS level and the decrease of total thiol concentration, catalase and SOD activities, being consistent with the results of former studies.^35,36^

Evidence from previous investigations indicated antioxidative properties of fenugreek.^37,38^ Our results also indicated that treatment with fenugreek seed extract and metformin had protective effects on oxidative damage by attenuating the TBARS level and augmenting the total thiol concentration, catalase and SOD activities.

The antioxidant effects of fenugreek in previous studies were considered on its flavonoid and polyphenol compounds ^37^. Furthermore, this plant contains various antioxidant substances, and its major antioxidant compounds are flavones of vitexin and isovitexin.^38^

Hyperglycemia and hyperlipidemia are common factors involved in the induction of ROS generation;^39,40^ therefore, the antioxidative property of fenugreek seed extract may be associated with its anti-hyperglycemic and anti-hyperlipidemia effects.

In DCM, the heart exhibits disordered arrangements of myocardial cells with increased mitochondrial damage, apoptosis, and fibrosis.^41^ In addition, it has been proved by excess production of inflammatory factors and reactive oxygen species, diabetes is an inflammatory disease. Cardiac inﬂammation causes increased expression of the vascular cell adhesion molecule (VCAM)-1(cell adhesion molecules [CAMs]) and intracellular adhesion molecule (ICAM)-1. CAMs, owing to their binding to its speciﬁc counter receptor. For example, the leukocyte and macrophage integrins mediate the transendothelial migration of immunocompetent cells into the cardiac tissue. These invading cells and cardiomyocytes produce proinﬂammatory cytokines such as interleukin (IL)-1, IL-18, and the tumor necrosis factor (TNF).^42^ These proinﬂammatory cytokines not only stimulate the expression of CAMs as a positive feedback mechanism, but also have direct and indirect cardiodepressive effects such as apoptosis via the intracellular serine-threonine kinase Akt pathway and modulation of cardiac function.^43^ Hyperglycemia worsens the mitochondrial generation of ROS and glucose oxidation, thereby causing DNA damage and contributing to accelerated apoptosis.^8^

We observed overexpression of the pro-apoptotic *Bax* gene, downexpression of the anti-apoptotic *Bcl2* gene as well as overexpression of the *ICAM1* gene expression, indicating increased apoptosis in STZ-diabetic myocardium, and being consistent with several previous reports implicating cardiomyocyte apoptosis in the pathogenesis of diabetic cardiomyopathy.^8,9^ *Bax* is one of the regulatory molecules in the apoptotic pathway acting as a pro-apoptotic protein. Upregulation of this gene expression in the cell, derives it to apoptosis by interacting with voltage-dependent anionic channels (VDAC) in the mitochondria membrane and activating them, leading to opening of these channels and loss of the membrane potential, thereby resulting in permeability transition and cytochrome C release into the cell cytoplasm.^44-46^ *Bcl2* could maintain the mitochondrial outer membrane integrity and prevent release of cytochrome c into the cytoplasm, thereby protecting against apoptosis.^9^

Moreover, hyperglycemia and hyperlipidemia in diabetes lead to increased oxidative stress and DNA damage, which is followed by activation of the P53 molecule, a nuclear regulating factor for the expression of genes related to cell survival and death that participate in diabetes-induced cardiac damage^47,48^ and regulate the expression of *Bax* and *ICAM1*.^49^ The prominent results of the current study demonstrated that in fenugreek extract and metformin treated groups, the gene expression of *Bax* and *ICAM1* was decreased, while *Bcl2* overexpression was observed. In addition, reduction of the apoptosis index in these groups was indicated. Therefore, this anti-apoptotic effect of fenugreek seed is in agreement with several similar previous reports.^50-52^ New investigations have revealed cardioprotective effect of this herb against apoptosis through intermediation in the renin-angiotensin signaling pathway.^16^ However, the apoptosis inducing feature of fenugreek seed in different models of cancer should be considered.^53-55^

Currently, metformin is the most widely used drug treatment for type 2 diabetes owing to its glucose-lowering effect and has been well-known for its potential relevance to cardiovascular diseases. This cardioprotective drug affects apoptosis and inflammation, which in diabetic cardiomyopathy is considered to be dysregulated.^56^ In our study, although metformin and different doses of fenugreek extract did not show salient hypoglycemic effects, reduction of the apoptosis index in the presence of these treatments was significant. These results may indicate that the anti-hyperglycemic and cardiovascular effects of anti-diabetic drugs could be exerted through various pathways, since the hypoglycemic effect of metformin has not been considerable, but it has higher cardiac protection in diabetes. Furthermore, the improved dose-dependent fenugreek seed extract results may be attributed to the higher amounts of its components in higher doses such as trigonellin and diosgenin steroids as well as the alkaloid and flavonoid contents of fenugreek seeds, which are responsible for the anti-inflammation, anti-apoptosis and anti-oxidant interesting activities of the plant^50-52^.

According to the acquired data, fenugreek, in addition to antidiabetic effects, indicated significant anti-oxidant and anti-apoptotic effects, as well as its effectiveness in reducing hyperlipidemia is much better than that of metformin.

Although the incidence of apoptosis was assessed only by measuring the expression of apoptosis-related genes, it is recommended that more evaluations be conducted at the protein level to better elucidate the effect of this plant on apoptosis in the course of diabetes. The ICAM1 marker, in addition to being associated with apoptosis, is an inflammatory factor. However, to investigate the anti-inflammatory effects of fenugreek in this model, it is necessary to study other related inflammatory signals and cytokines.

Nevertheless, since fenugreek has been traditionally used as an antidiabetic herbal medicine, more studies are necessary to further reveal the cardiovascular protective effects of this plant on diabetes through various mechanisms.

## Conclusion

Overall, the current study demonstrates that fenugreek seed extract may have high therapeutic potential in treating DCM and other cardiovascular disorders, through ameliorating metabolic abnormalities and oxidative stress, as well as regulating the genes involved in apoptosis

## Acknowledgments

This paper is extracted from a M.Sc. thesis in medical physiology by Soleyman Bafadam (Code A-1049). The authors would like to thank the Research Affairs Unit of Mashhad University of Medical Sciences for its financial support.

## Competing interest

The authors declare that there are no conflicts of interest.

## Ethical approval

The mentioned experiments were performed by observing animal welfare and protection laws, so that the Ethics Committee for Experimental Animals Use and Care (CEA) of the Mashhad University of Medical Sciences approved the protocol (approval No: 940828).

## Funding

Research Affairs Unit of Mashhad University of Medical Sciences(grant No. 940828).
